# Using Online Social Media for Recruitment of Human Immunodeficiency Virus-Positive Participants: A Cross-Sectional Survey

**DOI:** 10.2196/jmir.3229

**Published:** 2014-05-01

**Authors:** Patrick Yuan, Michael G Bare, Mallory O Johnson, Parya Saberi

**Affiliations:** ^1^Center for AIDS Prevention StudiesDepartment of MedicineUniversity of California, San FranciscoSan Francisco, CAUnited States

**Keywords:** HIV, AIDS, online social media, Facebook, Twitter, recruitment, Internet research, survey retention, online data collection software, non-financial incentives

## Abstract

**Background:**

There are many challenges in recruiting and engaging participants when conducting research, especially with HIV-positive individuals. Some of these challenges include geographical barriers, insufficient time and financial resources, and perceived HIV-related stigma.

**Objective:**

This paper describes the methodology of a recruitment approach that capitalized on existing online social media venues and other Internet resources in an attempt to overcome some of these barriers to research recruitment and retention.

**Methods:**

From May through August 2013, a campaign approach using a combination of online social media, non-financial incentives, and Web-based survey software was implemented to advertise, recruit, and retain participants, and collect data for a survey study with a limited budget.

**Results:**

Approximately US $5,000 was spent with a research staff designated at 20% of full-time effort, yielding 2034 survey clicks, 1404 of which met the inclusion criteria and initiated the survey, for an average cost of US $3.56 per survey initiation. A total of 1221 individuals completed the survey, yielding 86.97% retention.

**Conclusions:**

These data indicate that online recruitment is a feasible and efficient tool that can be further enhanced by sophisticated online data collection software and the addition of non-financial incentives.

## Introduction

Recruiting and retaining HIV-positive research participants is a crucial yet challenging endeavor [1-4]. Numerous studies indicate several unique challenges that prevent HIV-positive individuals from participating in research, such as self-presentation bias [5], access to adequate transportation [6], low socioeconomic status [7], and mistrust of research [5]. Although offering financial incentives can mitigate some of these factors, it can also incentivize fraudulent responses and repeat participation [8], which may diminish scientific validity. Moreover, HIV-positive individuals experience high levels of perceived stigma [5,9,10], elevated rates of depression [5,7,11,12], and increased risk of substance use. When combined, these issues create a formidable barrier to participation in research designed to examine these issues and decrease the generalizability of study findings.

In addition to these HIV-specific challenges, there are major impediments and compromises associated with research in general [13,14]. These include geographical and time constraints, labor to recruit and retain participants, limited financial resources, and logistical difficulties associated with survey administration or intervention conduction [14]. Failure to recruit and retain a sufficient number of eligible participants can be costly [15], threatens internal and external validity of the study [16], and, most importantly, deprives the scientific community of knowledge and potentially useful interventions [15,17].

Due to the near ubiquity of the Internet [18], it has become easier for people to engage in online surveys and research from the comfort and privacy of their own homes [19]. Specifically, online social media has been highly successful in capturing audiences [20]. Chief among these is Facebook, the second most popular website in the world [21]. With 198 million monthly active users and 93% of adult Internet users having a Facebook account, the average user spends approximately 1 out of every 8 minutes online or more than 11 hours per month on Facebook [22]. Twitter is another popular online social media platform, with over 49 million monthly active users [20]. These venues have already been used to successfully recruit research participants from various groups, such as mothers of advanced maternal age [23], individuals with Klinefelter’s syndrome [24], those with active depression [25,26], and smokers [27,28].

Therefore, if perceived stigma, cost of transportation, and physical distance are potential deterrents for HIV-positive individuals to participate in research, and time, geographical, and resource constraints are among the general difficulties of research, then Internet-based resources may be a logical solution to overcome many of these barriers. Thus far, the literature has indicated that online social media is ripe for researchers to use as a tool for recruitment [29,30]. By accessing online venues where potentially qualified participants are already spending time, it is possible to bridge the psychosocial and physical divides between research participants and researchers to achieve a successful outcome. Therefore, to capitalize on these existing resources, we used a novel combination of online social media along with non-financial incentives and contemporary data collection software for participant recruitment, retention, and data collection.

## Methods

### Study Design and Data Collection

We conducted a cross-sectional survey using online social media to recruit HIV-positive individuals. The purpose of the parent study was to examine the barriers to and facilitators of adherence to antiretroviral therapy and to describe the use of mobile telephones and other technologies to improve adherence among HIV-positive individuals living in the United States. Here, we describe the methods used for participant recruitment using online social media.

We implemented a campaign approach where participants were recruited through online social media such as Facebook, Twitter, and other social media platforms, such as LinkedIn, Craigslist, and Tumblr, from May 7 to August 31, 2013. The online survey was programmed using Qualtrics Research Suite (Qualtrics, Provo, UT), an online survey tool that allows researchers to build, distribute, and analyze online surveys in real time. The survey contained 112 items on demographics, HIV clinical outcomes, and use of technology. The University of California, San Francisco (UCSF) Committee on Human Research approved this study in April 2013.

### Participant Recruitment

A schedule was devised by the research assistant to regularly update and maintain these social media sites for a total of 8 hours per week (20% of full-time effort).

#### Facebook

##### Overview

We used 4 methods to recruit participants on Facebook: paid advertisements (ads), fan page, personal messages, and postings in groups.

##### Facebook Ads

A Facebook ad was created that ran separately and concurrently with the rest of the research recruitment campaign to further broaden exposure to the target population. With the assistance of a Facebook representative, a daily average budget of US $40 and a total budget of US $5,000 were established to cover the 4-month recruitment period. The average maximum bid per cost per click (CPC) was set at US $3.50, which was above the Facebook suggested bid range of US $0.52-$1.09, to outcompete other advertisements targeting the same groups. These advertisements were targeted at users with self-reported interests or “liked” topics in any of the following categories: HIV/AIDS; lesbian, gay, bisexual, transgender, queer, questioning, intersex, and asexual (LGBTQQIA); men who have sex with men (MSM); HIV co-morbidities such as tuberculosis and Hepatitis C; and unprotected sex.

A wide variety of advertisement pictures were chosen to cater to the target demographic. Pictures of inanimate or abstract objects, such as the AIDS red ribbon, were used as well as pictures of people representing various age ranges, genders, and sexual identities. Since banner advertisements targeting MSM of color have been shown to increase the click-through rate [31], we altered the racial/ethnic composition of the images. All of these advertisements used the banner title “Living with HIV?”, accompanied by a brief survey description (Multimedia Appendix 1). We obtained the rights to use stock photographs from various Internet sites.

We initiated the Facebook ad campaign with 3 advertisements but increased the number of advertisements to 5 in order to cater 2 advertisements specifically toward women and youth (18-29 years old). This decision was in response to the Facebook Ad Reports, which showed low levels of participation by women and youth. We varied ad pictures on a bi-weekly basis based on the number of Facebook ad clicks.

##### Facebook Fan Page

A Facebook fan page was created with the intention of recruiting participants and raising general awareness of the study. This was achieved by generating interesting and relevant posts for people living with HIV, which included news articles, study announcements, survey dissemination requests, pictures, videos, and HIV-related resources. Posted news articles related to HIV, medication adherence, and the use of technology as it pertained to HIV and medication adherence. On average, the research assistant updated the fan page every 3 to 4 days and posted about 2 new posts per day. A fan page description under the “About” section of the Facebook page was created to inform potential participants of the study and provide a link to the survey. A brief description of the study investigators and the organization was also included to build rapport and credibility with the audience. The research assistant interacted with other Facebook fan pages with similar purpose or interest by “liking” these organizations’ pages, which were found using keyword searches for HIV, AIDS, MSM, and health care.

##### Facebook Messages

The research assistant sent personalized Facebook messages on behalf of the study to community leaders, major HIV/AIDS organizations, and other related organizations, especially in states with low survey response rates. Survey response rates were estimated by examining Facebook Ad Reports. These messages contained information about the study, a link to the survey, and a request to repost the study link on the individual or organization’s corresponding Facebook page.

##### Facebook Groups

The research assistant also joined HIV-related Facebook groups to advertise for recruitment. These groups maintain Facebook pages based on real-life interests to facilitate discussions of relevant topics. Once requests to join these groups were granted, posts were made in the common thread requesting that interested participants click on the survey link.

#### Twitter

A Twitter account was created for the purpose of recruiting participants for the study and to continue building an online network to advertise for the survey. Through the study’s Twitter account (ie, “feed”), the research assistant followed any organization affiliated with topics such as HIV, AIDS, MSM, or global health care. Individuals were also followed if they were candid about their HIV seropositive status or being MSM, an HIV advocate, or a health educator.

Tweets were sent directly to both individuals and organizations as a request to re-tweet the survey link. Hashtags (#) related to the Twitter account were used to keep track of the number of times each message was tweeted and a shorter version of the study’s URL (ie, “tiny URL”) was generated to fit within the 140-character limit of tweets. The research assistant tweeted relevant content related to HIV/AIDS or interacted with other Twitter feeds by re-tweeting interesting and applicable topics on a weekly basis.

#### Other Social Media Platforms

##### LinkedIn

LinkedIn groups that included content related to HIV were specifically chosen to advertise to potential study participants. Postings regarding the study were made in groups that were created for people living with HIV, HIV advocates, community workers, and other researchers. Additionally, a request was made with each posting for group members to share the survey link with other potentially interested groups or individuals.

##### Craigslist

On a weekly basis, the research assistant posted advertisements containing the survey link and basic information about the study on nationwide Craigslist groups pertaining to MSM and health. Elements from the Touro 12-Step Process [32] were utilized, including guidelines on creating a thread title, generating an active and ongoing discussion, and being courteous.

##### Tumblr

A Tumblr blog was created and maintained with regular updates that mirrored the Facebook posts. Other blogs with content related to HIV were “liked” in order to build an audience and stimulate recruitment.

### Subjects and Research Engagement

Individuals who clicked on the study Web link were directed to the Qualtrics survey where they were required to consent online before being screened for eligibility. The survey was used to screen individuals based on the following inclusion criteria: age 18 years or older, HIV-positive serostatus, and currently living in the United States. We excluded individuals who had already taken the survey by limiting the number of survey attempts to 1 per Internet Protocol (IP) address and by asking participants if they had already taken the survey. Qualtrics was programmed to end the survey if the participant did not qualify. We ensured anonymity by not collecting any identifiers or personal health information and storing responses on encrypted and password-protected servers at UCSF.

Given the anonymous nature of the Web-based survey and lack of identifiers to verify repeat and fraudulent responses, monetary incentives were not offered in order to de-incentivize multiple survey attempts. However, in order to keep participants engaged, we motivated participants by inserting 5 medically interesting facts strategically throughout the survey titled “Fun Facts”, which were accompanied by visually stimulating and relevant pictures. Examples of these fun facts include: “Did you know that we exercise at least 30 muscles when we smile?” and “Did you know that your brain uses as much power as a 10-watt light bulb?” Additionally, to spark interest, we asked participants before beginning the survey if they knew of a natural substance that can be potentially effective against HIV. Upon completion of the survey, we embedded a link to a video about how bee venom is being studied by the Washington University School of Medicine as a potential drug to treat HIV [33].

We consulted the UCSF Center for AIDS Prevention Studies (CAPS) Community Advisory Board (CAB), which is comprised of stakeholders from Bay Area agencies and communities with the mission of channeling community input into the CAPS research agenda, providing advice on study methods, and improving recruitment methods. Based on the recommendations of the CAPS CAB, we modified the Facebook ad pictures to exclusively use photographs of people, the Facebook ad text to be more direct and motivating, and incorporated Tumblr as an additional online social networking venue for recruitment.

### Analysis

Three variables were created to estimate the relative value of each of the 3 main methods of recruitment: (1) “Facebook Page Interactions” to estimate user activity on the Facebook fan page, (2) “Twitter Interactions” to evaluate the overall activity on the Twitter feed, and (3) “Facebook Ad Clicks” to assess the overall success of the Facebook ad. The number of likes, comments, and shares on the Facebook page per recruitment day were extracted from Facebook Page Insights and summed to serve as an objective measure of Facebook Page Interactions. Data collected from Twitonomy (a Twitter analytics tool) included the number of retweets and mentions per recruitment day and were used to compute Twitter Interactions. Information on the number of clicks per day for the Facebook ad was obtained from Facebook Ad Reports. Using descriptive data from Qualtrics, we collected information on the number of survey clicks per day. We used Pearson’s pairwise correlation to examine the relationship between the number of survey clicks per day and Facebook Page Interactions, Twitter Interactions, and Facebook Ad clicks per day. Correlation values of 0 to .3 were deemed weak correlation, .3 to .7 indicated moderate correlation, and >.7 were considered strong correlation. The significance level of each correlation coefficient was also estimated. A *P* value of <.05 was deemed statistically significant.

As part of the survey, participants were also asked about their method of recruitment. These methods were categorized under one of the following categories: Facebook, Twitter, word-of-mouth, email from a listserv, and other (including LinkedIn, Craigslist, Tumblr, or other). We used 2-way frequency tables to examine the distribution of the method of recruitment based on participants’ age (categorized as 18-29.9, 30-39.9, 40-49.9, 50-59.9, and ≥60 years), race/ethnicity (White/Caucasian, African-American/Black, Latino, and other), male sex at birth, and sexual orientation (homosexual/gay, heterosexual, bisexual, and other). A chi-square test was used to assess the null hypothesis that the categorical variables are independent.

Last, to estimate research retention in the absence of monetary incentives, we compared the total number of participants who responded to the first survey question with the total number of respondents who answered the last survey question. All statistical analyses were conducted using Stata, version 13.1 (StataCorp, College Station, TX).

## Results

### Total Respondents

A total of 2034 individuals clicked on the survey Web link, averaging 18 clicks per day. Of those who clicked, 1977 people consented to take the survey, 1404 met the inclusion criteria and initiated the first question of the survey, and 1221 responded to the last question of the survey (86.97% retention). A total of 43 respondents answered affirmatively when asked if they had taken the survey before and were therefore excluded from the survey.

**Figure 1:**
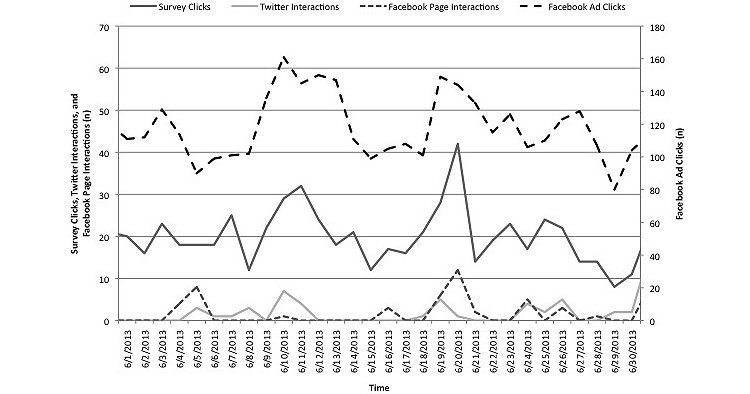
Number of Survey Clicks, Twitter Interactions, Facebook Interactions, and Facebook Ad Clicks in June 2013.

The study received a total of 10,006 Facebook Ad Clicks, 278 Facebook Page Interactions, and 161 Twitter Interactions during the recruitment period. Although each of these factors contributed to the total number of survey clicks per day, only the number of Facebook Ad Clicks was moderately correlated with the number of survey clicks per day based on the Pearson pairwise correlation (.52, *P*<.001). The Facebook Page Interactions and Twitter Interactions were weakly correlated with Facebook Ad Clicks (*r*=.17, *P*=.06; *r*=.18, *P*=.06, respectively). Figure 1 illustrates the number of survey clicks relative to the number of Facebook Ad clicks, Twitter Interactions, and Facebook Page Interactions for the month of June 2013. This serves to visually demonstrate the correlation between each of the 3 major methods of recruitment and the total number of survey clicks per day.

### Number of Survey Clicks, Twitter Interactions, Facebook Interactions, and Facebook Ad Clicks in June 2013

On average, participants spent 31 minutes to complete the survey. Table 1 encapsulates key demographics of participants by self-reported method of recruitment. According to these data, participants were primarily 40-49.9 years of age, identified as White/Caucasian, male at birth, and homosexual. Facebook was the most common method of recruitment across all reported demographics. With increase in age, participants were more likely to report being recruited through non-Facebook methods. Similarly, heterosexual individuals were more likely to report non-Facebook methods of recruitment compared to bisexual or homosexual participants. Email from a listserv was the second most commonly reported method for those aged 60 years or older and African Americans. Racial/ethnic categories were independent of self-reported method of recruitment.

A total of US $5,021 was spent on the Facebook ad (mean CPC = $0.64), which targeted a total of 14 million Facebook users and accumulated 1404 initiated surveys. Therefore, the estimated mean cost per survey initiation was approximately US $3.56. The reach of the Facebook ads included participants from 48 states plus the District of Columbia but did not include participants from either Alaska or Wyoming. From the Facebook fan page, the research assistant “liked” 490 other fan pages while 53 other organizations and individual Facebook users “liked” the study’s fan page. A total of 141 Facebook messages were sent to community leaders, organizations, and individuals requesting to have the survey link reposted.

Over the course of the recruitment period, the research assistant tweeted 572 times, received 199 Twitter followers, and followed 1092 Twitter feeds. In addition, the research assistant contacted a total of 16 LinkedIn groups, added 3 Tumblr sites, and posted on 3 Craigslist forums.

**Table 1:** Key participant demographics and self-reported method of recruitment.

Characteristic		Total, n	Facebook	Email from a Listserv	Word-of-mouth	Twitter	Other^a^	*P* value^b^
**Age, years, n (%)**								<.001
	18-29.9	246	211 (85.8)	7 (2.9)	15 (6.1)	2 (0.8)	11 (4.5)	
	30-39.9	274	233 (85.0)	11 (4.0)	21 (6.7)	5 (1.8)	4 (1.5)	
	40-49.9	508	422 (83.1)	29 (5.7)	36 (7.1)	6 (1.2)	15 (3.0)	
	50-59.9	474	381 (80.4)	37 (7.8)	33 (7.0)	5 (1.1)	18 (3.8)	
	≥60	402	256 (63.7)	55 (13.7)	31 (7.7)	23 (5.7)	37 (9.2)	
	Total^c^	1904	1503 (78.94)	139 (7.30)	136 (7.14)	41 (2.15)	85 (4.46)	
**Racial identification, n (%)**								.311
	White/Caucasian	995	802 (80.6)	67 (6.7)	81 (8.1)	11 (1.1)	34 (3.4)	
	Black/African American	126	97 (77.0)	14 (11.1)	9 (7.1)	2 (1.6)	4 (3.2)	
	Hispanic/Latino	181	157 (86.7)	8 (4.4)	9 (5.0)	2 (1.1)	5 (2.8)	
	Other	102	90 (88.2)	4 (3.9)	3 (3.0)	2 (2.0)	3 (2.9)	
	Total^c^	1404	1146 (81.62)	93 (6.62)	102 (7.26)	17 (1.21)	46 (3.28)	
**Sex at birth, n (%)**								<.001
	Male	1321	1102 (83.42)	76 (5.75)	86 (6.51)	14 (1.06)	43 (3.26)	
	Female	77	39 (50.7)	16 (20.8)	16 (20.8)	3 (3.9)	3 (3.9)	
	Total^c^	1398	1141 (81.62)	92 (6.58)	102 (7.30)	17 (1.22)	46 (3.29)	
**Sexual orientation, n (%)**								<.001
	Homosexual/Gay	1208	1007 (83.36)	73 (6.04)	80 (6.62)	11 (0.91)	37 (3.06)	
	Heterosexual	92	54 (58.7)	13 (14.1)	14 (15.2)	5 (5.4)	6 (6.5)	
	Bisexual/Other	91	75 (82.4)	6 (6.6)	8 (8.8)	1 (1.1)	1 (1.1)	
	Total^c^	1391	1136 (81.67)	92 (6.61)	102 (7.33)	17 (1.22)	44 (3.16)	

^a^Includes LinkedIn, Craigslist, and Tumblr.

^b^Based on chi-square test.

^c^Differences in number of responses for each characteristic are due to missing data; participants were not “forced” to respond to each demographic question.

## Discussion

### Principal Findings

Our study results indicate that the unique integration of online social media recruitment, Web-based survey, and non-financial incentives is an efficient and streamlined strategy for survey research with HIV-positive individuals. The restricted resources of US $5,000 in a 4-month recruitment timeframe with 20% research assistant’s time resulted in recruiting 1404 qualified participants in 48 states and the District of Columbia, averaging 18 survey responses per day, making this an efficient solution for participant recruitment. This is similar to other studies not focused on an HIV-positive population that have demonstrated the efficiency of Facebook ads [24,27]. Furthermore, we were able to access a difficult-to-reach population that may experience stigma and other barriers to research participation.

Even though Facebook has been used to effectively recruit HIV-positive individuals [34], our study is novel in that we utilized a campaign approach—using several different online social media platforms at once to benefit from various approaches and to maximize diversity. Compared to other methods of online recruitment, the Facebook ad was by far the most successful and least time consuming. The number of survey clicks per day and Facebook ad clicks was moderately correlated and highly statistically significant, illustrating the importance of a paid Facebook advertisement. We believe that setting a high CPC bid and increasing the number of interest groups as recommended by the Facebook representative, as well as using ad images of women and ethnic minorities [32] and more direct and motivating ad text as proposed by the CAPS CAB were associated with improved ad performance.

Although we observed a weak correlation between the number of survey clicks per day and both the Facebook Page and Twitter Interactions, these platforms were essential in establishing and maintaining a rapport with the study population and community. Such relationships were cultivated by liking and following relevant pages and groups of underrepresented populations, posting comments on walls and reposting relevant material, and sending personal messages. These techniques were particularly helpful given that the level of compliance with our requests was contingent upon the relationship and rapport between the researcher and the person or institution being contacted. For example, mass-produced messages and random posts on other Facebook pages or Twitter feeds were not as useful as personalized messages sent directly to a community leader or an HIV advocate with an established relationship. This highlights the social phenomenon known in economics as reciprocity, which correlates one’s likelihood of compliance with their familiarity with the one making the request [35]. Therefore, future researchers should not rely exclusively on online social media and underestimate the powerful nature of existing social networks to assist with recruitment efforts.

Retaining participants for Internet-based studies has been known to be difficult [36], particularly among African American and Latino MSM [31,37]. In response, our study catered ads for these demographics and implemented a novel incentive structure by adding interesting facts throughout the survey to motivate participants. We used this approach as opposed to one with pecuniary incentives to minimize duplicate and false responses. Given that the average response time for survey completion was 31 minutes and 86.97% of those who initiated the first question of the survey completed the survey, we believe this is a tactical approach that can be used in future online research with anonymous participants.

Since there are more Facebook users than any other social media site, it was not surprising that most participants identified Facebook as their primary method of recruitment. Moreover, the paid Facebook ad and the selection of targeted groups likely contributed to a higher percentage of Facebook recruits. However, it is unknown why older age and non-homosexual orientations were more likely to be associated with non-Facebook methods of recruitment. Future research should further examine possible reasons for these patterns. As such, the use of online social networks other than Facebook for recruitment remains a strong area of interest as venues for future research [38]. It is likely that sites such as LinkedIn, Craigslist, and Tumblr would be more useful for research within professional networks, other interest groups, or longitudinal research.

### Limitations

The self-reported demographics elucidated in Table 1 are not representative of the population of those living with HIV in the United States [39]. Responses revealed a higher proportion of individuals who identify as homosexual but fewer African Americans and Latinos. This may be attributed to HIV-specific reasons such as stigma, the necessity of having access to the Internet to participate in the survey, the need to be using social media to view recruitment advertisements, and the requirement for English proficiency to take the survey. Such drawbacks may be generalized to other study populations [24,40] and are important factors when considering the use of online social media for recruitment.

Another limitation of our study is the possibility of duplicate responses. A study using online social media also found this issue to be problematic, especially when participants were provided a financial incentive without the use of restrictive software [8]. However, attrition rates for online surveys in the HIV-positive MSM population are high [41], urging researchers to provide some form of incentive. With awareness of this issue, we implemented safeguards to protect the integrity of the survey data by introducing non-financial incentives, automatically disqualifying participants who used duplicate IP addresses, and asking participants whether they had already participated in the survey. This inquiry was a necessary precaution owing to the fact that 43 respondents admitted to taking the survey more than once. Future researchers might consider investigating the extent to which repeat online survey attempts are denied due to duplicate IP addresses using comparable online survey instruments and non-financial incentives. Even though we believe the likelihood of duplicate responses is low, it is not possible for us to determine if users circumvented these measures by accessing the survey through multiple devices or by providing inaccurate responses.

In terms of Facebook ad pictures, future research may be directed toward studying the effectiveness of different ad images by measuring the number of ad clicks and survey responses, which was a limitation of this study. Another limitation was that we were unable to verify the HIV serostatus of participants. However, given the lack of pecuniary incentives, it is unlikely that individuals would misrepresent themselves as being HIV-positive. Last, given that email from a listserv and directly contacting individuals using Facebook messages were valuable tools, it would have been beneficial for us to have asked participants about the specific listserv or the particular Facebook route (ie, Facebook ads vs personal message) in order to distinguish between various efforts.

### Conclusions

Online social media is an indispensable tool for recruiting participants because it has the potential to be cost-effective and efficient. Social media has the unique ability to transcend barriers to study recruitment such as physical distance, transportation, and limited time and financial resources. It also affords users the added benefits of privacy and anonymity, which may ameliorate the effects of perceived stigma in difficult to reach populations. Moreover, non-financial incentives in the form of trivia are a viable alternative to monetary incentives that yields high rates of retention while minimizing the chances of duplicate and fraudulent responses. Facilitating this process using online data collection software can further reduce cost and time associated with data collection. In sum, as Internet-based research tools become more practical, researchers must embrace these methods not only to maximize efficiency but also to enhance the scientific validity and generalizability of their findings. As such, online social media, alternative types of incentives, and Web-based survey software are well poised to become staple methods of recruitment and engagement in research.

## Multimedia Appendix 1

Facebook ad example.

## Abbreviations

CAPS CAB: Center for AIDS Prevention Studies Community Advisory Board
CPC: cost per click
IP: Internet Protocol
MSM: men who have sex with men
UCSF: University of California, San Francisco

## References

[1] Anastasi JK, Capili B, Kim GH, Chung A (2005). Clinical trial recruitment and retention of a vulnerable population: HIV patients with chronic diarrhea. Gastroenterol Nurs.

[2] McQuiston C, Uribe L (2001). Latino recruitment and retention strategies: community-based HIV prevention. J Immigr Health.

[3] Calsyn RJ, Klinkenberg WD, Morse GA, Miller J, Cruthis R, HIV/AIDS Treatment Adherence‚ Health Outcomes Cost Study Group (2004). Recruitment, engagement, and retention of people living with HIV and co-occurring mental health and substance use disorders. AIDS Care.

[4] Dodds S, Blakley T, Lizzotte JM, Friedman LB, Shaw K, Martinez J, Siciliano C, Walker LE, Sotheran JL, Sell RL, Botwinick G, Johnson RL, Bell D (2003). Retention, adherence, and compliance: special needs of HIV-infected adolescent girls and young women. J Adolesc Health.

[5] DiClemente RJ, Ruiz MS, Sales JM (2010). Barriers to adolescents' participation in HIV biomedical prevention research. J Acquir Immune Defic Syndr.

[6] Saberi P, Yuan P, John M, Sheon N, Johnson MO (2013). A pilot study to engage and counsel HIV-positive African American youth via telehealth technology. AIDS Patient Care STDS.

[7] Traeger L, O'Cleirigh C, Skeer MR, Mayer KH, Safren SA (2012). Risk factors for missed HIV primary care visits among men who have sex with men. J Behav Med.

[8] Quach S, Pereira JA, Russell ML, Wormsbecker AE, Ramsay H, Crowe L, Quan SD, Kwong J (2013). The good, bad, and ugly of online recruitment of parents for health-related focus groups: lessons learned. J Med Internet Res.

[9] Mahajan AP, Sayles JN, Patel VA, Remien RH, Sawires SR, Ortiz DJ, Szekeres G, Coates TJ (2008). Stigma in the HIV/AIDS epidemic: a review of the literature and recommendations for the way forward. AIDS.

[10] Sayles JN, Wong MD, Kinsler JJ, Martins D, Cunningham WE (2009). The association of stigma with self-reported access to medical care and antiretroviral therapy adherence in persons living with HIV/AIDS. J Gen Intern Med.

[11] Bing EG, Burnam MA, Longshore D, Fleishman JA, Sherbourne CD, London AS, Turner BJ, Eggan F, Beckman R, Vitiello B, Morton SC, Orlando M, Bozzette SA, Ortiz-Barron L, Shapiro M (2001). Psychiatric disorders and drug use among human immunodeficiency virus-infected adults in the United States. Arch Gen Psychiatry.

[12] Valente SM (2003). Depression and HIV disease. J Assoc Nurses AIDS Care.

[13] Maxine Patel, Victor Doku, Lakshika Tennakoon (2003). Challenges in recruitment of research participants. Advances in Psychiatric Treatment.

[14] Hulley SB (July). Designing Clinical Research.

[15] Nasser N, Grady D, Balke CW (2011). Commentary: Improving participant recruitment in clinical and translational research. Acad Med.

[16] Johnson MO, Dilworth SE, Neilands TB, Chesney MA, Rotheram-Borus MJ, Remien RH, Weinhardt L, Ehrhardt AA, Morin SF, NIMH HLP team (2008). Predictors of attrition among high risk HIV-infected participants enrolled in a multi-site prevention trial. AIDS Behav.

[17] Hogan JW, Roy J, Korkontzelou C (2004). Handling drop-out in longitudinal studies. Stat Med.

[18] Pew Internet & American Life Project (2013). Internet adoption.

[19] Zhang D, Bi P, Lv F, Tang H, Zhang J, Hiller JE (2007). Internet use and risk behaviours: an online survey of visitors to three gay websites in China. Sex Transm Infect.

[20] Business Insider (2013). Twitter has a surprisingly small number of US users.

[21] Alexa (2010). Top sites in United States.

[22] Hubspot 12 Essential Facebook Stats.

[23] O'Connor A, Jackson L, Goldsmith L, Skirton H (2014). Can I get a retweet please? Health research recruitment and the Twittersphere. J Adv Nurs.

[24] Close S, Smaldone A, Fennoy I, Reame N, Grey M (2013). Using information technology and social networking for recruitment of research participants: experience from an exploratory study of pediatric Klinefelter syndrome. J Med Internet Res.

[25] Morgan AJ, Jorm AF, Mackinnon AJ (2013). Internet-based recruitment to a depression prevention intervention: lessons from the Mood Memos study. J Med Internet Res.

[26] Meyer B, Berger T, Caspar F, Beevers CG, Andersson G, Weiss M (2009). Effectiveness of a novel integrative online treatment for depression (Deprexis): randomized controlled trial. J Med Internet Res.

[27] Ramo DE, Prochaska JJ (2012). Broad reach and targeted recruitment using Facebook for an online survey of young adult substance use. J Med Internet Res.

[28] Sadasivam RS, Volz EM, Kinney RL, Rao SR, Houston TK (2013). Share2Quit: Web-based peer-driven referrals for smoking cessation. JMIR Res Protoc.

[29] Ryan GS (2013). Online social networks for patient involvement and recruitment in clinical research. Nurse Res.

[30] Fenner Y, Garland SM, Moore EE, Jayasinghe Y, Fletcher A, Tabrizi SN, Gunasekaran B, Wark JD (2012). Web-based recruiting for health research using a social networking site: an exploratory study. J Med Internet Res.

[31] Sullivan PS, Khosropour CM, Luisi N, Amsden M, Coggia T, Wingood GM, DiClemente RJ (2011). Bias in online recruitment and retention of racial and ethnic minority men who have sex with men. J Med Internet Res.

[32] Ip EJ, Barnett MJ, Tenerowicz MJ, Perry PJ (2010). The Touro 12-Step: a systematic guide to optimizing survey research with online discussion boards. J Med Internet Res.

[33] Hood JL, Jallouk AP, Campbell N, Ratner L, Wickline SA (2013). Cytolytic nanoparticles attenuate HIV-1 infectivity. Antivir Ther.

[34] Mitchell JW, Petroll AE (2012). Patterns of HIV and sexually transmitted infection testing among men who have sex with men couples in the United States. Sex Transm Dis.

[35] Fehr E, Gächter S (2000). Fairness and retaliation: the economics of reciprocity. Journal of Economic Perspectives.

[36] Koo M, Skinner H (2005). Challenges of internet recruitment: a case study with disappointing results. J Med Internet Res.

[37] Khosropour CM, Sullivan PS (2011). Predictors of retention in an online follow-up study of men who have sex with men. J Med Internet Res.

[38] Van de Belt TH, Berben SA, Samsom M, Engelen LJ, Schoonhoven L (2012). Use of social media by Western European hospitals: longitudinal study. J Med Internet Res.

[39] Centers for Disease Control and Prevention (2013). HIV/AIDS.

[40] Eysenbach G, Wyatt J (2002). Using the Internet for surveys and health research. J Med Internet Res.

[41] Chiasson MA, Parsons JT, Tesoriero JM, Carballo-Dieguez A, Hirshfield S, Remien RH (2006). HIV behavioral research online. J Urban Health.

