# Supplementary figures and images for: Using Online Social Media for Recruitment of Human Immunodeficiency Virus-Positive Participants: A Cross-Sectional Survey
Source: J Med Internet Res. 2014 May 1;16(5):e117. doi: 10.2196/jmir.3229 (PMC4026571; doi:10.2196/jmir.3229)

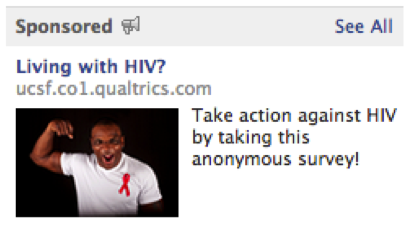

Supplement: Supplementary file 1 [file jmir_v16i5e117_app1.png]
